# How Should Treatment Decisions Be Made for Incapacitated Patients, and Why?

**DOI:** 10.1371/journal.pmed.0040035

**Published:** 2007-03-13

**Authors:** David I Shalowitz, Elizabeth Garrett-Mayer, David Wendler

Patients have gained unprecedented levels of control over their medical care, including prospective control over what treatments they will receive if they lose the ability to make decisions. The landmark *Cruzan* and *Quinlan* cases helped to secure patients' right to prospectively refuse life-sustaining treatments [1,2], while the Patient Self-Determination Act of 1990 mandated that patients be informed of the option to document their treatment preferences in an advance directive [3].

Despite these efforts, many individuals fail to complete an advance directive and seldom discuss their medical treatment preferences, often leaving clinicians with little indication for how they want to be treated [4–7]. Several groups have attempted to increase the number of people who complete an advance directive, and several approaches offer promise in this regard [8]. Yet, no method, no matter how exhaustive, will result in all individuals completing an advance directive, and no advance directive, no matter how detailed, will provide clear instructions for all clinical scenarios. For the foreseeable future, then, clinicians will continue to face the dilemma of how to treat some incapacitated patients in the absence of clear evidence regarding their treatment wishes.

Most states in the US direct clinicians to turn to surrogate decision makers, identified through standardized relationship hierarchies, to make treatment decisions for incapacitated patients who did not designate a surrogate while capacitated. Surrogates, whether designated by the patient or the state's standardized hierarchy, are instructed to make the treatment decision the patient would have made if capacitated. Under this “substituted judgment” standard, a surrogate asked to decide whether to initiate dialysis for a comatose patient, for example, should try to determine what decision the patient would have made in the circumstances.

Individuals often fail to complete an advance directive.

The medical profession's reliance on surrogates to make treatment decisions for incapacitated patients gains support from the fact that the relevant considerations for choosing a substitute decision maker all seem to point to the family and loved ones. Allowing the patient's family and loved ones to make treatment decisions seems to respect their status as vital individuals in the patient's life [9–11] and may provide comfort at a difficult time. Reliance on those who know the patient best also seems to promote patient autonomy by maximizing the chances that the incapacitated patient's preferences will guide what treatments they receive. Finally, current practice seems consistent with patients' preferences for how treatment decisions should be made [12–14]. To test whether, in fact, these considerations all support the same approach, we assessed whether a population-based decision aid might predict patients' treatment preferences more accurately than surrogates.

## Identifying a Potential Alternative to Surrogates

Recent data suggest that basing treatment decisions on how individuals similar to the patient want to be treated might predict patients' treatment preferences more accurately than patients' surrogates [15–17]. To pursue this possibility, we asked whether a population-based method that predicts which treatment patients want based on the treatment preferences of individuals similar to the patient might make more accurate predictions than patient designated and next of kin surrogates. We call this method a “population-based treatment indicator.”

Imagine that a 70-year-old Native American male with a PhD and severe Alzheimer disease develops pneumonia. To decide whether to administer antibiotics, clinicians first look to any evidence of the patient's treatment preferences. When the patient's treatment preferences are unknown, current practice is to ask the patient's designated or next of kin surrogate to predict, based on their knowledge of the patient, whether he would want antibiotics. The population-based treatment indicator, in contrast, predicts whether the patient would want antibiotics based on the preferences of individuals similar to the patient in similar circumstances.

Implementation of this approach would involve the clinician entering the incapacitated patient's circumstances—pneumonia and severe Alzheimer disease—and his characteristics—older, well educated, Native American, male—into a computer. The treatment indicator's database analyzes the treatment preferences of similar individuals and estimates the likelihood that the patient would want antibiotics to treat his pneumonia. Physicians could then use this information to help them make a treatment decision. A finding that 90% of highly educated Native American men over the age of 50 do not want to receive antibiotics to treat pneumonia in the setting of advanced Alzheimer disease would provide strong evidence that this patient would not want antibiotics in his circumstances.

This approach will require data on which characteristics influence individuals' medical treatment preferences. Does gender predict whether individuals want to receive antibiotics in the setting of severe Alzheimer disease? Does religion? Ancestry? Whether one was born before or after the *Cruzan* and *Quinlan* cases? Characteristics associated with different treatment preferences, along with their probabilities, would be entered into the database of the population-based treatment indicator. As more data are collected, additional associations would be entered, thus allowing the indicator to cover more circumstances, and make more accurate treatment predictions.

The population-based treatment indicator does not assume that all individuals of a given gender, race, or religion have the same medical treatment preferences. Rather, the indicator simply incorporates all and only those factors which the data show are associated with particular medical treatment preferences, along with the strength of these associations. The members of any given group, no matter how narrowly defined, will invariably have different treatment preferences in some cases. For this reason, physicians sometimes will make mistakes when relying on a population-based treatment indicator to predict the treatment preferences of incapacitated patients. Recognizing the inevitability of mistakes, the present analysis asks whether a population-based treatment indicator predicts patients' treatment preferences more accurately than surrogates. This assessment involves three steps: 1) estimate the predictive accuracy of surrogates; 2) define a preliminary population-based treatment indicator based on the currently available data regarding individuals' treatment preferences; and 3) determine the predictive accuracy of the preliminary population-based treatment indicator.

## How Accurate Are Surrogates?

The best way to assess how accurately surrogates predict patients' treatment preferences would be to determine how closely surrogates' treatment decisions match patients' preferences at the time patients are incapacitated. Because there is no way to determine what medical treatments patients want at the time they are incapacitated, most empirical studies assess how accurately surrogates predict patients' treatment choices in hypothetical scenarios.

A recent article systematically analyzed the 16 empirical studies published in English that used this methodology [18]. Each study posed hypothetical scenarios in which the patient would be unable to make medical decisions. Patients were asked whether, in these scenarios, they would want to receive various medical interventions, such as ventilation and cardiopulmonary resuscitation (CPR). Each surrogate was then independently asked to predict what choices the patient made in the included scenarios. The 16 analyzed studies presented a total of 151 hypothetical scenarios to 2,595 surrogate–patient pairs, collectively analyzing 19,526 paired patient–surrogate responses. More than 90% of the 151 scenarios involved an intervention necessary to save or sustain the patient's life. For example, one study posed the following scenario [19]:

**You recently suffered a major stroke leaving you in a coma and unable to breathe without a machine. After a few months, the doctor determines that it is unlikely that you will come out of the coma. If your doctor had asked whether to try to revive you if your heart stopped beating in this situation, what would you have told the doctor to do?**

Analysis of the 16 studies reveals that surrogates accurately predict patients' treatment preferences approximately 68% of the time.

The empirical data also suggest that the two most frequently endorsed methods for improving surrogates' predictive accuracy—reliance on surrogates designated by the patient rather than the default next of kin, and explicit discussion of patients' treatment preferences—are ineffective. In the studies conducted, surrogates designated by the patient were no more accurate than surrogates appointed according to the relevant state's “next-of-kin” relationship hierarchy. Similarly, explicit discussion of patients' treatment preferences did not increase surrogates' ability to predict patients' treatment choices. Finally, several studies find that physicians are even less accurate than surrogates at predicting patients' treatment preferences [20–22].

## Developing a Population-Based Treatment Indicator

To identify data for a preliminary population-based treatment indicator, we searched the existing published literature for empirical data on individuals' medical treatment preferences. This search identified a few relevant studies. Polling results suggest patients' preferences for receiving a given intervention are correlated with their predicted post-intervention health state [23,24]. When it is predicted that the patient will return to an acceptable health state, the majority of patients want to receive most interventions. When it is predicted that the patient will end up in an unacceptable health state, the majority of patients decline most interventions.

These findings raised the question of which health states individuals regard as acceptable, and which they regard as unacceptable. Further research found that most Americans consider being permanently in a coma or otherwise permanently unable to reason, remember, or communicate as no better than, or even worse than death [8,25,26], and would not want lifesaving interventions in these situations [27–29]. For example, one national survey reported that 75% of 1,311 adults would not want to be kept alive if they were in a coma with no chance of recovery [27]. Data also suggest that many Americans want life-saving interventions when there is at least a 1% chance that the treatment will lead to what they consider an acceptable health state [30].

Putting these findings together, we defined a preliminary population-based treatment indicator which predicts that a given patient will want life-saving treatment when there is at least a 1% chance, following the intervention, that the patient will reach a health state which includes the ability to reason, remember, and communicate. Conversely, the preliminary population-based treatment indicator predicts that patients will not want life-saving treatments when there is greater than a 99% chance that, following the treatment, they will be left permanently unable to reason, remember, or communicate.

The data we identified concern the treatment preferences of US citizens in general, and do not provide information on the characteristics of individuals who have different treatment preferences. For example, the data show that most Americans would not want life-saving treatment if they would be left unable to reason, remember, or communicate. Based on this finding, our preliminary population-based treatment indicator predicts that a given patient would not want life-saving treatment if they were in a coma. While this prediction matches the preferences of the majority of Americans, it fails to identify Americans who would want life-saving treatment even in the presence of a coma. Collection of additional data to identify the characteristics of these individuals would yield a more accurate treatment indicator.

## Comparing Surrogates to a Population-Based Treatment Indicator

To determine the predictive accuracy of our preliminary population-based treatment indicator, we evaluated its accuracy in the scenarios used by the extant empirical studies to evaluate surrogate accuracy. We first selected all the scenarios that provided both surrogates' percentage predictive accuracy and the percentage of patients who wanted the offered intervention. The accuracy of the treatment indicator was calculated by determining the percentage of cases in which the prediction of the population-based treatment indicator would match the patient's treatment preference. This accuracy was then compared to the percentage of surrogates who accurately predicted the patient's treatment preferences.

Scenarios were excluded from this comparison if they 1) did not involve a life-saving intervention; 2) did not describe the proposed intervention or the patient's predicted post-intervention health state; 3) included a post-intervention health state that is not addressed by the current data on which health states the US general public finds acceptable; or 4) did not provide data on patients' preferences for receiving the proposed intervention. For a complete list of scenarios included in the comparison, see Table 1.

**Table 1:**
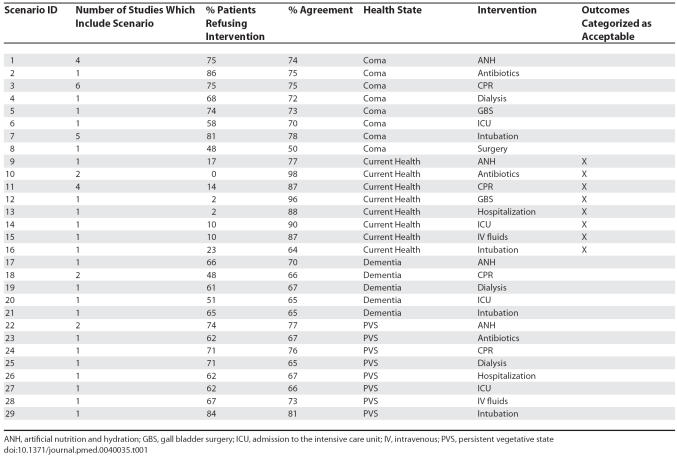
Scenarios Included in the Surrogate/Treatment Indicator Comparison

Many of the scenarios used in the empirical studies do not provide *quantitative* estimates of patients' prognoses. One scenario asks patients whether they would want various lifesaving interventions if it was “unlikely” they would come out of a coma or if “no one can be certain what [the patient's] level of functioning would be if she ever did come out of the coma [31].” To provide the most complete test possible, we defined the preliminary population-based treatment indicator to predict that patients do not want life-saving interventions when the likelihood of recovery or the predicted health outcome are described in negative terms. Conversely, we defined it to predict that patients want life-saving interventions when the likelihood of recovery and the predicted health outcome are described in positive terms.

Analysis reveals that this very preliminary population-based treatment indicator predicts patients' treatment preferences with essentially the same accuracy as surrogates (Table 2). Overall, the treatment indicator correctly predicted the patient's treatment preferences in 78.5% of cases, while, in these same scenarios, surrogates correctly predicted the patient's treatment preferences in 78.4% of cases. We hypothesize that the accuracy of surrogates in these scenarios is inflated relative to the review's overall findings (i.e., 78% versus 68%) because many of the scenarios selected for comparison were “easier” than the excluded scenarios, involving the patient's current health and hypothetical health states and interventions with which the general public is familiar, such as coma and CPR.

**Table 2:**
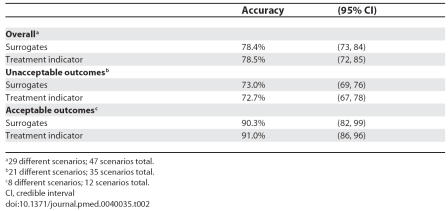
Predictive Accuracy of Surrogates Versus a Preliminary Population-Based Treatment Indicator

## Refining the Population-Based Treatment Indicator

Our preliminary population-based treatment indicator is limited, in several ways, by the existing data. First, our preliminary version is based on the preferences of the US general public. The present data did not allow us to define a preliminary indicator that takes into account the fact that certain groups, especially cultural and religious groups, systematically have different preferences for medical treatment at the end of life compared to the general population [32]. Second, the present data did not allow us to incorporate the fact that the characteristics of individual patients, including age and gender [33], and aspects of the patient's post-intervention health state, beyond the ability to reason, remember, and communicate, often influence whether they want to receive medical treatments. Patients may want to be treated when there is essentially any chance of returning to complete health, but insist on a higher chance of success when the predicted health outcome includes a serious disability, such as complete paralysis of the lower limbs.

Third, data indicate that individuals' preferences for receiving medical interventions are influenced by the characteristics of the interventions themselves [34]. Patients are more likely to decline invasive procedures (e.g., intubation) compared to less invasive procedures (e.g., oral medications). Given the current paucity of systematic data, we were unable to incorporate these considerations in our preliminary treatment indicator.

While the preliminary treatment indicator likely could be made more accurate by incorporating data on these factors, the two most widely endorsed methods for improving surrogate accuracy—values discussions and patient-designation of surrogates—appear ineffective [18]. Moreover, current estimates, which are based on hypothetical scenarios, may represent an overestimate of surrogates' true predictive accuracy. Surrogates may be comparatively less able to process the medical facts relevant to making treatment decisions for incapacitated patients in actual cases. And surrogates may be less able to reason clearly when faced with the stress, sorrow, and uncertainty that accompany caring for loved ones at the end of life.

## Using the Population-Based Treatment Indicator

When the treatment preferences of an incapacitated patient are unknown, the clinician would enter the patient's characteristics and circumstances into the database of the population-based treatment indicator. The indicator provides a statistical prediction of which treatment option the patient would want based on the treatment preferences of similar patients in similar situations. In practice, this prediction could be used in at least three ways.

First, the treatment prediction might supplement the surrogate decision-making process. In this approach, treatment decisions for incapacitated patients would still be made in consultation with the patient's surrogate. The population-based treatment indicator would be used to help diminish uncertainty surrounding the patient's wishes by identifying the treatment preferred by similar patients. For example, when the patient's preferences are unknown, it likely would be useful for physicians and surrogates to know that 90% of individuals like the patient want to be intubated in the patient's situation. This approach might help to relieve some of the burdens associated with making decisions for incapacitated patients, while allowing family and loved ones to retain final decision-making authority.

More radically, the population-based treatment indicator could be used in place of surrogate decision makers. When the patient's treatment preferences are unknown, treatment decisions would be based on the prediction of the population-based treatment indicator. Finally, competent individuals could be asked to indicate on their advance directives whether, in the event of incapacity, they prefer to have treatment decisions made by a designated surrogate, or based on the population-based treatment indicator. Since not all individuals complete advance directives this method would have to be supplemented with a default approach.

## Who Should Make Treatment Decisions for Incapacitated Patients?

The fact that a preliminary treatment indicator is as accurate as surrogates, and the accuracy of the treatment indicator could be increased with the collection of more systematic data, suggests that a refined population-based treatment indicator likely would predict patients' treatment preferences more accurately than surrogates. This conclusion reveals that the most important considerations do not all point to the family and loved ones as the appropriate decision makers for incapacitated patients. While allowing intimate others to have a say in the care of incapacitated patients supports current reliance on surrogates, accuracy in matching treatment decisions to patients' preferences supports the use of a population-based treatment indicator.

To determine which approach clinicians should adopt, it will be necessary to conduct additional research. Normative analysis will be needed to prioritize the relevant considerations: what is more important, which treatment decisions get made, or who makes them? Ideally, this analysis should be informed by empirical data on the preferences of individuals and family members, as well as data on the impact that making end-of-life treatment decisions has on family and loved ones. To what extent does making decisions for incapacitated patients allow families and loved ones to process a tragic event, and to what extent does it represent an unwelcome burden at a time of extraordinary stress? The few studies to directly assess the impact of decision making on surrogates do not indicate whether making treatment decisions is, overall, beneficial or burdensome [35–37].

Future studies might compare the benefits and burdens surrogates experience when they make treatment decisions versus the impact of using a population-based treatment indicator. A finding that family members experience important benefits would provide an important reason to continue to rely on surrogates. A finding that family members do much better if clinicians rely on population-based treatment aids would provide an important reason to consider pursuing this approach.

We do not know why surrogates are frequently inaccurate when predicting their charges' treatment preferences. More research should consider this issue and whether it might be possible to identify other ways to improve surrogate accuracy. Finally, US states have their own laws regarding how treatment decisions should be made for incapacitated patients. Changes in the method of making treatment decisions for incapacitated patients may require changes to these laws.

## Conclusion

Current reliance on surrogates gains support from the fact that the four most important considerations—respect for family and loved ones, impact on family and loved ones, respect for patients' treatment preferences, and respect for patients' preferences regarding who makes treatment decisions for them—all seem to point to the patient's family and loved ones as the appropriate decision makers for incapacitated patients. However, systematic analysis suggests that a preliminary population-based treatment indicator, one based on currently available data, predicts patients' preferences as accurately as surrogates, and implies that a refined treatment indicator which took into account additional data on individuals' treatment preferences likely would predict incapacitated patients' preferences more accurately than surrogates.

This conclusion reveals that identification of a best practice requires prioritization of the relevant considerations regarding who should make treatment decisions for incapacitated patients. Normative analysis on this question should be informed by empirical data. Do patients care more about who makes decisions for them, or what decisions are made? Does making end-of-life treatment decisions benefit or burden families and loved ones overall?

## Abbreviations

CPR: cardiopulmonary resuscitation
